# Recrudescence of incontinentia pigmenti presenting as a paraneoplastic syndrome: A natural experiment of NF-kB blockade in an inflammatory malignancy

**DOI:** 10.1016/j.jdcr.2023.07.036

**Published:** 2023-08-11

**Authors:** Blake Elizabeth Brooks, Leslie Robinson-Bostom, Cathy Massoud

**Affiliations:** a Department of Dermatology, St. Luke’s University Health Network, Bethlehem, Pennsylvania; b Department of Dermatology, Warren Alpert Medical School of Brown University, Providence, Rhode Island; c Department of Dermatology, Richmond Veterans Affairs Medical Center, Richmond, Virginia

**Keywords:** incontinentia pigmenti, NF-kB, medical dermatology, oncology, paraneoplastic, TNF-α

## Introduction

Incontinentia pigmenti (IP)—an X-linked–dominant genodermatosis—results from NF-kB essential modulator/inhibitor kappa kinase (IKK)-gamma gene mutations, rendering the cells unable to activate the NF-kB pathway and highly susceptible to TNF-induced apoptosis.^1^ Various clinical findings affect the skin, hair, teeth, nails, eyes, and central nervous system. Blaschkoid skin lesions demonstrate 4 stages: inflammatory, verrucous, hyperpigmented, and hypopigmented/atrophic. Cutaneous progression is attributed to hyperproliferation and necrosis of IKK-gamma-deficient cells with subsequent release of proinflammatory cytokines by neighboring IKK-gamma-positive cells, enhancing apoptosis and resulting in the burnout of disease in infancy.^1^^,^^2^ Adult patients may demonstrate residual swirled to reticulated hyperpigmentation. Rare cases of cutaneous IP recrudescence after infancy may result from the stimulation of residual IKK-gamma-deficient cells that previously evaded apoptosis, and TNF-α is a potential underlying trigger.^2^

## Case report

**Fig 1:**
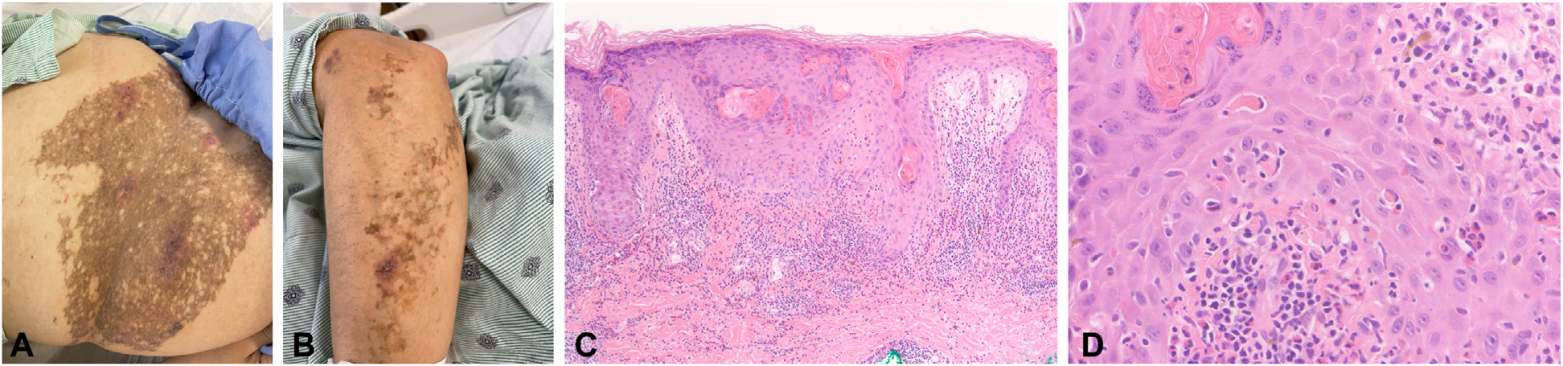
Cutaneous findings of a woman with paraneoplastic incontinentia pigmenti recrudescence. Right hip/flank (**A**) with agminated vesicles and left forearm (**B**) with vesicles and hyperkeratotic plaques overlying chronic reticulated hyperpigmentation (**A**). Right hip tangential biopsy demonstrating eosinophilic spongiosis with dyskeratosis and dermal melanophages (*arrow heads*), consistent with both vesicular and hyperpigmented stages of incontinentia pigmenti (Hematoxylin-eosin stain; original magnifications: **C**, ×100; **D**, ×400).

A 35-year-old woman with a history of IP complicated by epilepsy, monocular blindness, conical teeth, and family history of IP (mother, sister, and daughter) presented with epigastric pain status after recently treated *Helicobacter pylori* infection. She was afebrile with otherwise negative review of systems but had profound anemia (hemoglobin = 5.9 g/dL). Esophagogastroduodenoscopy and imaging demonstrated gastric adenocarcinoma without any evidence of metastatic disease. The dermatology department was consulted for a pruritic eruption present since admission. Cutaneous examination revealed agminated vesicles and hyperkeratotic plaques overlying reticulated hyperpigmentation (Fig 1, *A*, *B*). The results of polymerase chain reaction studies for lesional herpes simplex/varicella zoster virus were negative. Right hip biopsy revealed eosinophilic spongiosis with dyskeratosis and melanophages (Fig 1, *C*, *D*); biopsy of the left forearm (pathology not pictured) revealed hyperkeratosis, papillomatosis, dyskeratosis, and vesiculation. The patient reported 2 similar cutaneous eruptions in adolescence during febrile viral illnesses. Clinicopathologic correlation led to a diagnosis of paraneoplastic IP recrudescence as the patient was afebrile and without viral symptoms or recent immunizations.

Topical corticosteroids (triamcinolone 0.1% and clobetasol 0.5% creams) reduced blistering and pruritus within 3 days. Vesicles resolved, but the irritation overlying hyperpigmented patches persisted during neoadjuvant FOLFOX (folinic acid, fluorouracil, oxaliplatin) chemotherapy. Despite malignancy treatment response based on imaging, 5 months after admission, gastrectomy with lymphadenectomy demonstrated adenocarcinoma with lymphovascular/perineural invasion and positive lymph nodes. Postoperatively, no further skin irritation was noted, and CT findings for chest/abdomen/pelvis were negative for metastatic disease status after adjuvant chemotherapy. The patient remains without disease or skin lesion recurrence 15 months after resection.

## Discussion

Cases of cutaneous IP recrudescence in the setting of viral infections and immunizations have been reported in the literature. Recrudescence is thought to be caused by TNF-α-induced stimulation of residual IKK-gamma-deficient cells that are unable to activate the NF-kB pathway and are consequently vulnerable to TNF-induced apoptosis.^2^ Here, we report a case of paraneoplastic IP recrudescence occurring in the setting of *H. pylori*-associated gastric adenocarcinoma—a malignancy associated with elevated levels of TNF-α.^3^ This novel association was noted in the oldest patient who demonstrated IP recrudescence in the English medical literature.

The NF-kB pathway is an attractive oncologic therapeutic target as its inhibition theoretically impedes survival signals in malignant cells.^4^ In nonmutated cells, NF-kB remains inactive in the cytoplasm through its association with IkB. In response to stimuli, IkB is phosphorylated by IKK, allowing degradation of IkB and nuclear translocation of NF-kB. Current NF-kB pathway therapeutic targets include inhibition of upstream IKK, inhibition of IkB proteasomal degradation, and repression of NF-kB binding to DNA.^5^ IP serves as a natural model of NF-kB blockade through upstream IKK inhibition, demonstrating sensitivity of cells to apoptosis when exposed to proinflammatory cytokines. Furthermore, paraneoplastic IP recrudescence due to gastric adenocarcinoma emphasizes the potential for directed IKK-γ-deficient cell apoptosis in the presence of malignancy-induced TNF-α signaling.

## Conflicts of interest

None disclosed.
